# Characteristics and local risk factors of community-acquired and health-care-associated *Staphylococcus aureus* pneumonia

**DOI:** 10.1038/s41598-022-23246-1

**Published:** 2022-11-04

**Authors:** Li-Na Lee, Wen-Ru Chou, Jann-Yuan Wang, Yen-Liang Kuo, Chi-Yueh Chang, Yi-Chien Lee, Shao-Hsien Tung, Wen-Ching Tsao, Ke-Yun Chao, Wei-Lun Liu

**Affiliations:** 1 Department of Laboratory Medicine, Fu Jen Catholic University Hospital, Fu Jen Catholic University, New Taipei City, Taiwan; 2 Internal Medicine, Fu Jen Catholic University Hospital, Fu Jen Catholic University, New Taipei City, Taiwan; 3 School of Medicine, College of Medicine, Fu Jen Catholic University, No. 510, Zhongzheng Rd., Xinzhuang Dist., New Taipei City, 24205 Taiwan; 4 Department of Laboratory Medicine, National Taiwan University College of Medicine and Hospital, Taipei, Taiwan; 5 Department of Internal Medicine, National Taiwan University College of Medicine and Hospital, Taipei, Taiwan; 6 Department of Respiratory Therapy, Fu Jen Catholic University Hospital, New Taipei City, Taiwan; 7 Department of Critical Care Medicine, Fu Jen Catholic University Hospital, Fu Jen Catholic University, New Taipei City, Taiwan; 8 Data Science Center, College of Medicine, Fu Jen Catholic University, New Taipei City, Taiwan

**Keywords:** Diseases, Risk factors

## Abstract

This study aims at identifying characteristics, risk factors and mortality of community-acquired (CAP) and health-care-associated pneumonia (HCAP) by *Staphylococcus aureus* (*S*. *aureus*). We retrieved adults with *S. aureus* CAP or HCAP diagnosed by blood or pleural effusion culture in 2.6 years, and compared with those of *Streptococcus pneumoniae* (*S. pneumoniae*) CAP or HCAP diagnosed by blood or respiratory culture, or urine antigen. We found 18 patients with CAP and 9 HCAP due to *S. aureus* (female 33%, 66.6 ± 12.4 years-old), and 48 patients with CAP and 15 HCAP due to *S pneumoniae* (female 41%, 69.5 ± 17.5 years). Diabetes mellitus (52% vs. 24%, *p* = 0.019), hemodialysis (11% vs. 0%, *p* = 0.046), skin lesions (44% vs. 0%, *p* < 0.001), cavitary nodules (37% vs. 1.6%,* p* < 0.001) and pleural effusions (48% vs. 18%, *p* = 0.007) were more common in staphylococcal than pneumococcal group. Three patients with staphylococcal pneumonia had acute myocardial infarction. Pneumonia severity index (139 ± 52 vs. 109 ± 43, *p* = 0.005) and 30-day mortality (41% vs. 9.5%, *p* = 0.001) were higher in staphylococcal group. Multivariate analysis showed underlying disease (especially cancer and cirrhosis), risk class 4/5, altered mentality, shock and bilateral pneumonia were risk factors for 30-day mortality.

## Introduction

Severe communityacquired pneumonia (CAP) caused by *Staphylococcus aureus* (*S. aureus*) has been reported since 2005, following the influenza season of 2003–2004 and 2006–2007 in the United States (USA)^1–3^. Among the initially reported cases many occurred in young adults without underlying diseases but with preceding influenza, and most were caused by methicillin-resistant *S. aureus* (MRSA). *S. aureus* was later reported to be an important cause of health-care-associated pneumonia (HCAP)^4^.

However, clinical features of CAP or HCAP caused by *S. aureus* varied in studies from different regions. The average age of patients with staphylococcal CAP was 45, 49, and 60 years in studies from Brazil^5^, Canada^4^, USA^6^, respectively. The mortality of *S. aureus* CAP was 0%, 13% and 46%, in reports from Canada, USA, and Australia, respectively^4,6–8^. Preceding influenza was a risk factor for primary staphylococcal pneumonia in an earlier series^2^, but not in a prospective study published later that identified chronic hemodialysis as a risk factor for MRSA CAP^6^. Therefore, clinical characteristics and risk factors for staphylococcal CAP/HCAP may differ among regions. To help first-line physicians diagnose this potentially fatal pneumonia, we conducted this study to investigate characteristics and risk factors of *S. aureus* CAP and HCAP in New Taipei City (a densely populated industrial city in Northern Taiwan) and compared them with those of CAP/HCAP caused by *Streptococcus pneumoniae* (*S. pneumoniae*).

## Results

### Patients number and diagnostic methods

During the period of 2 years and 7 months, we identified 27 adult patients with staphylococcal CAP (*n* = 18) or HCAP (*n* = 9). All had *S. aureus* isolated from blood (Table S1, supplementary materials). Two (7%, both having CAP) also had *S. aureus* cultured from pleural fluid, 11 (41%) sputum, 2 (7%) nasopharynx and 7 (26%) non-respiratory sites including wound and urine. All the 27 patients (except for one who was found comatose on roadside and sent to our ER) had respiratory symptoms at presentation, suggesting primary staphylococcal pneumonia. The one with initial coma had lobar pneumonia in the right lower and patchy consolidation in right upper lobe, liver cirrhosis, prolonged prothrombin time and cerebral hemorrhage. Thus all 27 patients were diagnosed to have primary staphylococcal pneumonia. Image study found that chest radiographs and computed tomography (CT, performed in 12 or 44%) of the 27 patients all showed patchy or lobar consolidation with or without nodules, compatible with primary staphylococcal pneumonia with or without secondary septic emboli.

We identified 63 adult patients with pneumococcal CAP (*n* = 48) or HCAP (*n* = 15) during the same period. Sixty one (97%) has blood culture performed at ER or within 48 h after admission. Only four (6%) had positive blood culture of *S. pneumoniae* (Table S2, supplementary materials), of whom one also had positive culture from sputum and another one cerebral spinal fluid (CSF). The other 59 cases were diagnosed on positive sputum culture and pneumococcal antigen (*n* = 2) or positive pneumococcal antigen alone (*n* = 57). All the 63 patients had respiratory symptoms at presentation, suggesting primary pneumococcal pneumonia. Chest radiographs and CT (performed in 16 or 25%) of the 63 patients all showed lobar or patchy consolidation with or without mottling shadows, compatible with primary pneumococcal pneumonia.

### Clinical characteristics

Table 1 lists clinical characteristics. Patients with staphylococcal pneumonia were all middle aged or old. However, some patients with pneumococcal CAP were young and healthy (six or 9.5% < 40 year-old). Both staphylococcal and pneumococcal pneumonia occurred year-round, although more common in cool season. Co-detection of other pathogens, including influenza, was observed in 11% of staphylococcal and 44% of pneumococcal pneumonia (*p* = 0.435). MRSA accounted for 13 (48%) of staphylococcal pneumonia. Underlying diseases were present in 26 (96%) and 21 (67%) patients with staphylococcal and pneumococcal pneumonia, respectively (*p* = 0.007). Diabetes mellitus (DM) and chronic hemodialysis were more common in the staphylococcal than pneumococcal group (52% vs. 24%, *p* = 0.018 and 11% vs. 0%, *p* = 0.042, respectively). Among patients with staphylococcal CAP, two abused intravenous drugs.

**Table 1:** Characteristics of patients with community-acquired or health-care-associated pneumonia caused by *Staphylococcus aureus* or *Streptococcus pneumoniae.*

Characteristics	*S. aureus* (N = 27)	*S. pneumoniae* (N = 63)	*p*
Age (y)	66.6 ± 12.4 [42–83]	69.5 ± 17.5 [33–99]	0.437
Female	9 (33)	26 (41)	0.637
Case No. per month: cold season (Nov–Apr)	19/18 (1.1)	43/18 (2.4)	
Case No. per month: warm season (May–Oct)	8/13 (0.6)	20/13 (1.5)	
**Co-detection of other pathogens**	2 (7)	16 (25)	0.051
Influenza	1 (4)	12(19)	0.098
*Mycoplasma pneumoniae*	1 (4)	2 (3)	> 0.999
Current or ex-smoker	17 (63)	31 (49)	0.323
Alcoholism	4 (15)	2 (3)	0.111
**Co-morbidity**	26 (96)	42 (67)	0.007
Cancer	7 (26)	11 (17)	0.522
Diabetes mellitus	14 (52)	15 (24)	0.018
Cerebrovascular/neurodegenerative disease	6 (22)	13 (21)	0.860
CKD without hemodialysis	4 (15)	7 (11)	0.857
CKD under regular hemodialysis	3 (11)	0	0.042
Liver cirrhosis	3 (11)	5 (8)	0.960
COPD	11 (41)	17 (28)	0.338
Symptom duration (days)	2.1 ± 1.7	2.3 ± 2.0	0.807
Altered mental status	8 (30)	8 (13)	0.096
Shock	6 (22)	10 (16)	0.692
SPO_2_ (%)	86.9 ± 10.2	91.8 ± 8.0	0.099
**Skin lesions at presentation**	12(44)	0	< 0.001
Cellulitis/diabetic foot	5 (19)	0	
Scabies	3* (11)	0	
Other lesions or intravascular devices	6^#^ (22)	0	
**Chest X-ray/CT: multi-lobe infiltrates**	23 (85)	45 (71)	0.253
Multiple cavitary nodules	10 (37)	1 (1.6)	< 0.001
Pleural effusion	13 (48)	11 (17)	0.005
Pneumonia severity index	139 ± 52 [42–232]	109 ± 43 [23–221]	0.005
Risk class IV or V	23 (85)	40 (63)	0.067
Intubation	6 (22)	12 (19)	0.969
30-day mortality, No. (%)	11 (41)	6 (9.5)	0.001
**Duration of admission (days)**			
Alive at discharge	24.2 ± 16.6 [7–62]	12.8 ± 10.5 [2–53]	0.001
Succumbed	12.3 ± 11.3 [1–29]	12.8 ± 10.4 [1–28]	0.819

CKD, chronic kidney disease; COPD, chronic obstructive pulmonary disease; CT, computed tomography.

Data are presented as number (percent), means ± standard deviations, or [ranges].

*All had diabetes. Two were on chronic hemodialysis.

^#^Including bed sores (*n* = 3), intravascular devices (*n* = 3), carbuncle (*n* = 1), and herpes zoster (*n* = 1); some patients had more than one type of skin lesions.

The two groups had similar clinical manifestations. The presenting oxygen saturation was lower in the staphylococcal group. The most obvious differences in physical signs were skin lesions, including cellulitis, scabies, wound infection, herpes zoster, pressure sores, carbuncles or intravenous devices, at presentation in 11 of the staphylococcal, but none of the pneumococcal group (41% vs. 0%, *p* < 0.001). All those with skin lesions were diabetic.

Chest radiographs and CT scans revealed that pleural effusions (48% vs. 17%, *p* = 0.005) and multiple cavitary nodules (37% vs. 1.6%, *p* < 0.001, Fig. S1, supplementary materials) were more common in patients with staphylococcal than pneumococcal pneumonia.

### Pneumonia severity index and mortality

Average PSI was significantly higher in the staphylococcal group. Totally 85% and 63% of the staphylococcal and pneumococcal group, respectively, presented with a PSI risk class of ≥ 4 (*p* = 0.067). Patients with staphylococcal pneumonia had significantly higher 30-day mortality than those with pneumococcal pneumonia (41% vs. 9.5%, *p* = 0.001). Patients with methicillin-susceptible *S. aureus* (MSSA) and those with MRSA pneumonia had similar PSI (148 ± 54 vs. 137 ± 45, *p* = 0.582) and 30-day mortality (50% vs. 31%, *p* = 0.555). Among 30-day survivors, the average admission duration was 24.2 and 12.8 days for those with staphylococcal and pneumococcal pneumonia, respectively (*p* = 0.001).

### Complication

Table S3 lists major complications. Pyothorax that required catheter/chest tube/ thoracotomy occurred in two patients in each group; however, pyo-pneumothorax was observed only in two patients with staphylococcal pneumonia. Acute myocardial infarction (AMI) occurred in 3 patients with staphylococcal pneumonia (all men with MSSA, Table S4). Two of them had AMI at presentation with shock. The third one developed AMI simultaneously with cerebral artery emboli on the 27th day of admission. Two had underlying chronic kidney disease (CKD). All the three patients presented to ED with thrombocytopenia, prolonged activated thromboplastin time (APTT) and increased international normalized ratio (INR), suggestive of disseminated intravascular coagulation (DIC). All three died within 2 days of AMI.

### Empirical antimicrobials

Empirical antimicrobials used at ED for patients with staphylococcal pneumonia were mostly β-lactam-β-lactamase inhibitor (BLBLI) combinations, including piperacillin tazobactam and cefoperazone sulbactam, second-generation cephalosporin, and combination of antimicrobials that often included vancomycin or teicoplanin (Table S5, supplementary materials). Seven (26%) did not receive effective antimicrobials within 48 h of presentation and had a higher 30-day mortaIity than the 20 who received effective antimicrobials within 48 h (57% vs. 35%, *p* = 0.567). By contrast, empirical antimicrobials used for patients with pneumococcal pneumonia were cefoperazone sulbactam, fluoroquinolone, piperacillin tazobactam and amoxicillin clavulanate, without vancomycin, teicoplanin, or any combinations including them. Seven (11%) did not receive antimicrobials at ED, but all were discharged uneventfully after 5–18 days.

### Risk factors for mortality

**Figure 1:**
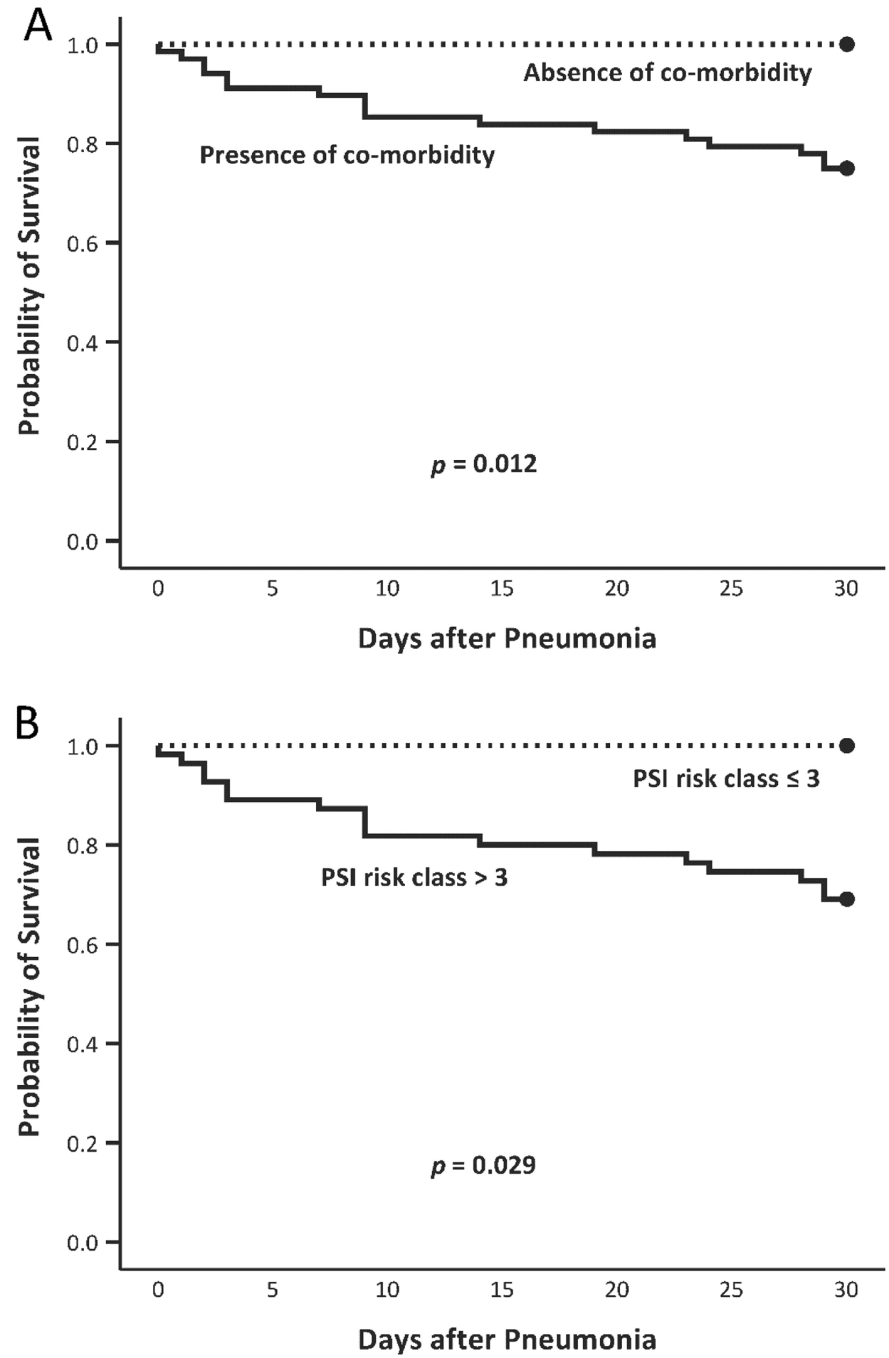
(**A**) Survival curves for patients with pneumonia caused by *Staphylococcus aureus* or *Streptococcus pneumoniae* with and without comorbidities (total: 90 cases, 22 without comorbidities [dotted line] and 68 with comorbidities [solid line]). (**B**) Survival curves for the patients with comorbidities (total: 68 cases, 13 with risk classes of 1–3 [dotted line] and 55 with risk classes of 4 or 5 [solid line]).

We analyzed risk factors associated with 30-day mortality among all the 90 patients with pneumonia (due to either *S. aureus* or *S. pneumoniae*) using multivariate regression analysis. First we discovered that all patients without co-morbidity (*n* = 22) survived. Therefore, THE presence of comorbidity is the most crucial prognostic factor for mortality (log-rank test *p* = 0.012; Table 2, Fig. 1A). After subtracting these 22 cases we analyzed risk factors among the remaining 68 patients with co-morbidity, and found that all the 13 patients with PSI risk classes 1–3 survived. Therefore, PSI risk class of 4 or 5 was the second most crucial prognostic factor for mortality (Table 2, Fig. 1B). After excluding 13 patients with risk classes of 1–3, we analyzed risk factors among the remaining 55 patients with co-morbidity and risk class of 4 or 5 using Cox regression backward selection model. Variables analyzed were pathogenic organisms, infection type (CAP or HCAP), age (≤ or > 65 years), sex, smoking status, blood culture result, DM, cancer, heart failure, chronic obstructive pulmonary disease (COPD), CKD with or without hemodialysis, cirrhosis, CVA, bilateral infiltrates, multiple cavitary nodules, shock, mental status, tachypnea, PaO_2_ (≥ or < 60 mm Hg), skin lesions, and effective antimicrobial use within 3 days. We identified five additional risk factors for 30-day mortality: cancer, cirrhosis, altered mental status, shock, and bilateral lung lesions (Table 3).

**Table 2:** Univariate Kaplan–Meier analysis of the effect of comorbidities on the 30-day mortality rate of the 90 patients with pneumonia and of the effect of risk class on the 30-day mortality rate of the 68 patients with comorbidities.

Population	Factor	Status	Total No	Death	Mortality rate (%)	p*
Overall (N = 90)	Co-morbidity	Present	68	17	25	0.012
		Absent	22	0	0	
Patients with co-morbidity (N = 68)	Risk class	≤ 3	13	0	0	0.029
		> 3	55	17	31	

*p value was calculated using the log-rank test.

**Table 3:** Risk factors for 30-day mortality in patients with pneumonia caused by *Staphylococcus aureus* or *Streptococcus pneumoniae* with comorbidities and pneumonia severity index risk classes of 4 or 5 (*n* = 55), as determined through Cox proportional hazards regression analysis.

Risk factor	*p* value	Hazard ratio	95% confidence interval
Cancer	< 0.001	32.18	4.91–211.07
Liver cirrhosis	< 0.001	100.06	8.80–1137.39
Altered mental status	< 0.001	55.10	8.43–360.03
Shock	0.001	7.99	2.36–27.05
Bilateral lung lesions	0.017	6.38	1.40–29.03

## Discussion

Our study found that staphylococcal CAP and HCAP occurred throughout the year in our community, with a prevalence lower than pneumococcal pneumonia. Influenza virus has been identified as a potential predisposing factor for staphylococcal CAP^1–3^. Because of the retrospective nature of this study, influenza testing was not performed for all the patients. Nevertheless, the low rate of influenza co-infection among patients who underwent influenza testing (1/11, 9%) in our study was similar to that reported in a prospective study from USA (8.1%)^6^, indicating that staphylococcal pneumonia can occur with or without preceding influenza.

We reviewed only adults in this study. Our patients with staphylococcal pneumonia were middle-aged or old, mostly with underlying diseases, in contrast to a previous study in which half of the patients with staphylococcal CAP were < 18 year-old and healthy^3^. The age range of our staphylococcal patients also differed from that of the pneumococcal group, in which 10% were aged < 40 years and healthy. The absence of patients with no underlying disease in the staphylococcal group suggested that certain condition predisposed hosts to *S. aureus* pneumonia. DM, chronic hemodialysis and skin lesions were more common in the staphylococcal group, similar to previous studies from Brazil, USA and Australia^5–7^. Patients with these underlying diseases had higher risk of staphylococcal pneumonia because: (1) diabetic or chronic hemodialysis patients are prone to *S. aureus* colonization in nose and skin, which increases the risk of *S. aureus* infection^9,10^. (2) Skin sores, scabies and intravascular lines (present in 44% of our staphylococcal but none of pneumococcal group) were risk factors for invasive staphylococcal infection^7^. Three of the patients with staphylococcal pneumonia and DM (two were receiving hemodialysis) had scabies at presentation. Scabies is associated with overcrowding and poor hygiene^11^. In Taiwan it is associated with bed-ridden status, nursing home residence and catheters^12^. Because scabies can be treated with scabicides and by cleaning bedding and clothing, early diagnosis of scabies may reduce the risk of staphylococcal pneumonia.

Bilateral cavitary nodules (37%) and pleural effusions (48%) were more common among our patients with staphylococcal pneumonia, similar to previous studies^4,13^. The association of rapidly progressive cavitating pneumonia with a preceding influenza due to Panton–Valentine leukocidin (PVL) produced by MRSA or MSSA has been reported^14^. We did not perform PVL assays and could not verify this association and our patients were elderly with negative influenza antigen or PCR. Nevertheless, the mortality was equally high in our study.

Three of fatal patients with staphylococcal pneumonia had AMI. Laboratory data suggested DIC in two. *Staphylococcus aureus* septicemia has been known to induce platelet and coagulation activation, fibrinolysis, thrombosis and DIC^15^, but AMI has not been previously reported to be associated with staphylococcal pneumonia. Whether AMI is related to DIC with thromboembolism in the coronary system remains unclear, given that all the three patients had risk factors for coronary artery disease, including smoking, DM, and CKD.

The average PSI score and 30-day mortality of patients in the staphylococcal group were higher than the pneumococcal group, similar to previous report^6^. The mortality was even higher (57%) among those without effective antimicrobials in 48 h. For the pneumococcal group, the 30-day mortality was 9.5%, similar to data from a USA (12.5%)^16^ and an Australian study (11.1%)^7^, and the seven patients in pneumococcal group without antimicrobial treatment at ED survived. The observation suggests higher virulence of *S. aureus* than *S. pneumoniae*, and poorer host immunity of patients who contracted staphylococcal pneumonia. Another factor that may contribute to the lower PSI score and mortality in the pneumococcal group was the percentage of bacteremia, which was 100% (27/27) for the staphylococcal and 6% for the pneumococcal group. Fifty-nine (94%) of the 63 patients in the pneumococcal group was diagnosed by positive urinary antigen; among them 57 had negative blood culture. The low rate of positive culture in patients with pneumococcal pneumonia may be due to the tendency of *S. pneumoniae* to autolyze when reaching the stationary phase of growth and antibiotic treatment prior to specimen collection^17^. Nevertheless, previous studies revealed that patients with pneumococcal pneumonia and positive pneumococcal bacteremia had higher^18^ or similar^19^ 30-day mortality than those without bacteremia. In our study patients with pneumococcal pneumonia and bacteremia had 30-day mortality rate (0/4) similar to non-bacteremic patients (6/59 or 10.2%, *p* = 0.834), and on multivariate analysis bacteremia was not a significant risk factor. We need to perform a prospective, larger study to see if bacteremia contributes to mortality.

Multivariate analysis showed risk factors for 30-day mortality were underlying diseases, risk class 4/5, cancer, cirrhosis, presenting with altered mental status, shock and bilateral pneumonia. A previous study found hemoptysis, erythroderma and leukopenia were associated with mortality in young patients (median age 14.5 years) with CAP due to PVL-producing *S. aureus*^20^. In a recent study of *S. aureus* CAP (age 29–67 years), MRSA and PVL were risk factors of mortality^21^. For pneumococcal pneumonia, age, smoking, alcohol abuse, solid tumor, liver and renal diseases were predictors of mortality^22^.

Our study has limitations: (1) Because of the retrospective nature, not all patients underwent testing for coexisting pathogens. Whether influenza was associated with staphylococcal or pneumococcal pneumonia was unclear. (2) We did not examine PVL that may help explain the virulence and complications associated with staphylococcal pneumonia. (3) Case number was small.

In conclusion, Staphylococcal CAP/HCAP in our community mainly affected elderly patients with DM or CKD. Many had skin lesions or intravenous catheters. AMI was a lethal complication. 30-day mortality in the staphylococcal group (41%) was higher than the pneumococcal group (9.5%), especially among those without effective antimicrobials in 48 h (57%). Risk factors for 30-day mortality for all patients with staphylococcal or pneumococcal pneumonia were underlying disease, risk class 4/5, cancer, cirrhosis, altered mentality, shock, and bilateral pneumonia.

## Material and methods

### Patients and methods

This study was conducted in Fu Jen Catholic University hospital, a primary-care hospital opened in October 2017 in New Taipei city with 460 beds currently. The study was approved by the ethics committee of Fu Jen Catholic University Hospital (FJUH108004), and the requirement of the informed consent was waived for the study. We defined *S. aureus* as the causative organism of pneumonia if it was isolated from blood or pleural fluid^23^. From the electronic database of our microbiology laboratory, we retrieved adult patients (> 17 years-old) with *S. aureus* isolated from blood or pleural fluid between October 2017 and April 2020, and reviewed their electronic records. We then identified all cases that fulfilled criteria for CAP or HCAP. Patients who presented to our emergency (ED) or outpatient department with at least 2 of the following criteria were defined as having CAP: fever, chest pain, cough, shortness of breath, in addition to having radiographic pneumonia, and without any criteria for HCAP^23,24^. Patients were determined to have HCAP if they had been hospitalized within 90 days; lived in nursing home; or had received intravenous antibiotics, chemotherapy, wound care, or hemodialysis in health-care facility within 30 days^25^. We calculated their pneumonia severity index (PSI) score and risk of death within 30 days^26^.

For comparison, we also retrieved adult patients with diagnosis of pneumococcal CAP or HCAP during the same period. We defined *S pneumoniae* as the causative organism of pneumonia if it was isolated from blood or pleural fluid, or if pneumococcal antigen was detected in urine^23^. We compared their clinical data and images with those of patients with staphylococcal CAP or HCAP.

### Microbiological and viral testing

All patients had blood culture sampled at ED or within 48 h of admission, except for two patients with *S. pneumonia*e CAP. Blood culture was performed using Virtuo BacT/ALERT system (Biomerieux, Hazelwood, MO, USA). Staining and culturing of samples for common bacteria/fungi or *Mycobacterium tuberculosis*, urinary antigen tests for *S. pneumoniae* and *Legionella pneumophila* (BinaxNow, Alere, USA), nasopharyngeal swab for influenza antigen, influenza polymerase chain reaction (PCR) (Luminex ARIES system, Austin, TX, USA) or SARS-CoV-2 real-time PCR (Applied Biosystems, Waltham, MA, USA) were performed if deemed necessary by the attending physician. Bacterial identification and antimicrobial susceptibility were performed using VITEK 2 system (VITEK 2XL, Biomerieux, Duram, NC, USA).

### Statistical analysis

Quantitative data were expressed as mean ± standard deviation, and differences between groups were analyzed by *t*-test. Percentages of cases with certain characteristics were compared using chi-square test. Associations between 30-day mortality and various factors were analyzed using chi-square test and multivariate logistic regression model. A two-sided *p* < 0.05 was considered significant. All analyses were performed using SAS (Version 9.2, SAS Institute Inc., Cary, NC).

### Statement of ethics

We conducted the trial in accordance with good clinical practice guidelines and the Declaration of Helsinki. The study was approved by the Institutional Review Board of the Fu Jen Catholic University Hospital, New Taipei City, Taiwan (FJUH108004). The informed consent was waived because of the retrospective nature of this study.

## Supplementary Information

Supplementary Information.

## Data Availability

Data collected and analyzed in the current study are available from the corresponding author on reasonable request.
