# Supplementary material for: Characteristics and local risk factors of community-acquired and health-care-associated Staphylococcus aureus pneumonia
Source: Sci Rep. 2022 Nov 4;12:18670. doi: 10.1038/s41598-022-23246-1 (PMC9636242; doi:10.1038/s41598-022-23246-1)

**Supplementary materials**

**Table S1. Positive culture samples of 27 patients with community-acquired pneumonia (CAP) or health-care-associated pneumonia (HCAP) caused by *Staphylococcus aureus*.**

| **Sample with positive *S aureus* culture** | **No.(%) of CAP patients (n=18)** | **No.(%) of HCAP patients (n=9)** | **Total No.(%) (n=27)** |
| --- | --- | --- | --- |
| Blood only | 6 (33) | 4 (44) | 10 (37) |
| Blood, pleural fluid and sputum | 1 (5) | 0 | 1 (4) |
| Blood, pleural fluid and urine | 1 (5) | 0 | 1 (4) |
| Blood and sputum | 5 (28) | 2 (22) | 7 (26) |
| Blood, sputum and non-respiratory sites | 1 (5) | 1 (11) | 2 (7) |
| Blood, nasopharynx and non-respiratory sites | 1 (5) | 0 | 1 (4) |
| Blood, sputum, nasopharynx and non-respiratory sites | 1 (5) | 0 | 1 (4) |
| Blood and non-respiratory sites | 2 (11) | 2 (22) | 4 (4) |
| Total | 18 | 9 | 27 |

**Table S2. Results of cultures and pneumococcal antigen tests for 63 patients with CAP or HCAP caused by *Streptococcus pneumoniae*.**

| **Blood culture results** | **Other culture results** | **Urine antigen** | **No. of episodes** | | |
| --- | --- | --- | --- | --- | --- |
|  |  |  | **CAP** | **HCAP** | **Total** |
| *S pneumoniae* | Sputum: *S pneumoniae* | Positive | 1 | 0 | 1 |
| *S pneumoniae* | CSF: *S pneumoniae* | Negative | 1 | 0 | 1 |
| *S pneumoniae* | Not done | Not done | 0 | 1 | 1 |
| *S pneumoniae* | Not done | Positive | 0 | 1 | 1 |
| No growth | Sputum: *S pneumoniae* | Positive | 2 | 0 | 2 |
| No growth | Sputum culture not done or results not decisive | Positive | 42 | 13 | 55 |
| Blood culture not done | Not done | Positive | 2 | 0 | 2 |
| Total |  |  | 48 | 15 | 63 |

**Table S3. Complications in patients with pneumonia caused by *Staphylococcus aureus* or *Streptococcus pneumoniae.***

| **Complication** | ***S. aureus***  **(n=27)** | ***S. pneumonia*e (n=63)** | ***p*** | **Mortality (%)** |
| --- | --- | --- | --- | --- |
| Pyothorax | 2 (7) | 2 (3) | 0.751 | 0 |
| Pyopneumothorax | 2 (7) | 0 | 0.165 | 100 |
| Acute myocardial infarction | 3 (11) | 0 | 0.042 | 100 |
| Stroke (hemorrhage or embolic) | 2 (7) | 0 | 0.165 | 100 |
| Infective endocarditis | 1 (4)* | 0 | 0.595 | 0 |
| Septic arthritis | 1 (4) | 0 | 0.595 | 100 |
| Meningitis | 0 | 1 (2) | 0.666 | 0 |

***** The patient used morphine.

**Table S4. Clinical and laboratory characteristics of three patients with acute myocardial infarction as a complication of pneumonia caused by *Staphylococcus aureus*.**

|  | No. 6 | No. 14 | No. 27 |
| --- | --- | --- | --- |
| Age(Y)/Sex | 82/M | 68/M | 82/M |
| Month | May | August | May |
| CAP or HCAP | CAP | CAP | HCAP |
| Smoking | Current smoker | (-) | Ex-smoker |
| Drinking | Social | (-) | (-) |
| Underlying disease | Hepatitis C, gout | Diabetes mellitus, hypertension, CKD,HBV | Lung cancer, valvular heart disease, CKD, T- spine surgery |
| Time of AMI | At presentation | Day 27 | At presentation |
| Shock | At presentation | At presentation | Not observed |
| Lab data at ER |  |  |  |
| Leukocyte (10^3^ cells/uL) | 6.06 | 12.85 | 18.79 |
| Platelet (10^3^ cells/uL) | 63 | 74 | 28 |
| Creatinine (umol/L) | 114.9 | 654.2 | 344.8 |
| APTT (sec) | 53 | 57.4 | 57.2 |
| INR | 1.24 | 2.02 | 2.22 |
| Fibrinogen (umol/L) [N: 6.03-11.00] | 7.09 | 6.53 | NA |
| FDP (ug/ml) [normal: < 4.6] | 71.5 | 11.4 | NA |
| D-dimer (ng/dL) [normal <500] | >10,000 | 2112 | NA |
| MRSA or MSSA | MSSA | MSSA | MSSA |
| Source of positive SA culture | Blood, joint fluid, nasopharynx, | Blood, pleural fluid, urine | Blood |
| Complications other than MI | Shock, respiratory failure | Shock, pyo-pneumothorax, cerebral embolism | Respiratory failure |
| Intubation | Yes | Yes | No (palliative care) |
| Under effective antibiotic(s) | Yes | Yes | Yes |
| Survival time after admission | 2 days | 28 days | 20 hours |

Abbreviations: APTT, activated partial thromboplastin time; CAP, community-acquired pneumonia; CKD, chronic kidney disease; AMI, acute myocardial infarction; ER, emergency room; FDP, fibrin degradation product; HCAP, health-care-associated pneumonia; INR: international normalized ratio; MRSA, methicillin-resistant *Staphylococcus aureus*; MSSA, methicillin-susceptible *Staphylococcus aureus*; SA, *Staphylococcus aureus*; NA, not available.

**Table S5. Antimicrobials used at the emergency or outpatient department to treat patients with CAP or HCAP caused by *Staphylococcus aureus* or *Streptococcus* *pneumoniae*.**

| **Antimicrobials** | ***S. aureus* No. (%)** | ***S. pneumoniae* No. (%)** |
| --- | --- | --- |
| Fluoroquinolone | 0 | 14 (22) |
| Amoxicillin and clavulanate | 0 | 8 (13) |
| 2^nd^-generation cephalosporin | 5 (19) | 7 (11) |
| 3^rd^-generation cephalosporin | 1 (4) | 1 (1.5) |
| Piperacillin tazobactam | 7 (26) | 10 (16) |
| Cefoperazone sulbactam | 7 (26) | 15 (24) |
| Imipenem cilastatin | 0 | 1 (1.5) |
| Combination: | 7 (26) | 0 |
| 2^nd^-generation cephalosporin + piperacillin tazobactam | 1 | 0 |
| 2^nd^-generation cephalosporin + teicoplanin | 1 | 0 |
| 3^rd^-generation cephalosporin + vancomycin | 1 | 0 |
| Meropenem + vancomycin | 1 | 0 |
| Meropenem + fluoroquinolone | 1 | 0 |
| Piperacillin tazobactam + fluoroquinolone | 1 | 0 |
| Vancomycin + fluoroquinolone | 1 | 0 |
| No antimicrobials at ER | 0 | 7 (11) |
| Total | 27 (100) | 63 (100) |

Fig. S1. Figure S1A shows chest radiology of an 82 year-old man with community-acquired pneumonia caused by *Staphylococcus aureu*s at presentation to ED, with faint small nodular and patchy shadows. He was in shock and respiratory failure. Figure S1B shows chest CT immediately after intubation, with cavitary nodules in bilateral apical region. Blood, sputum, nasopharyngeal swab and knee joint fluid all grew methicillin-susceptible *Staphylococcu*s *aureus*.

**A**


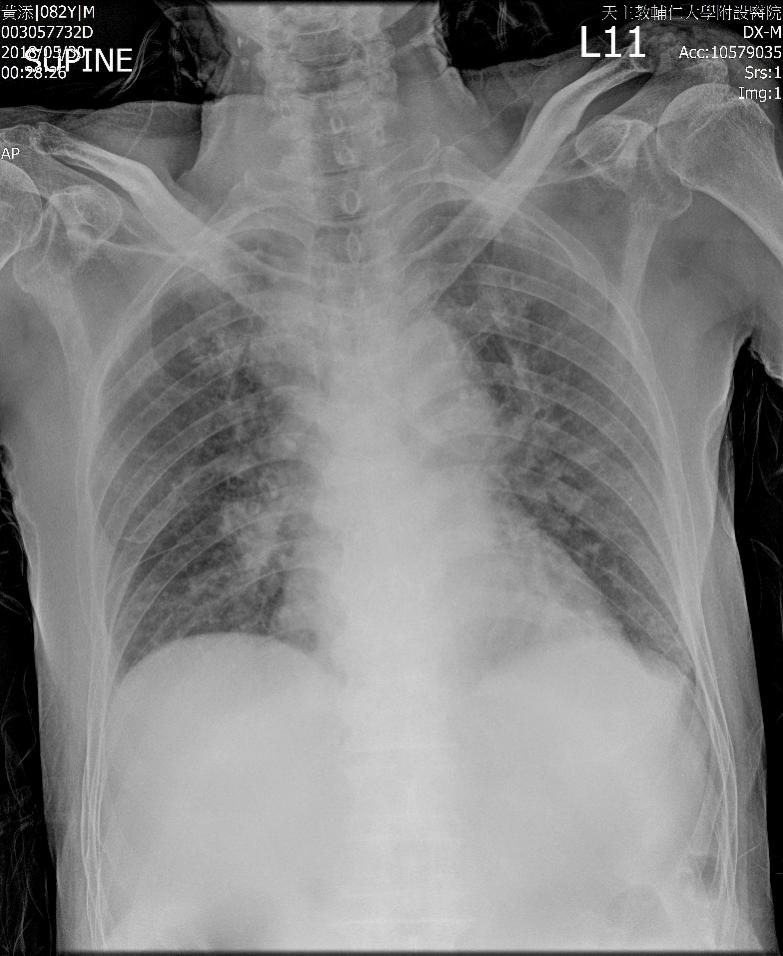


**B**


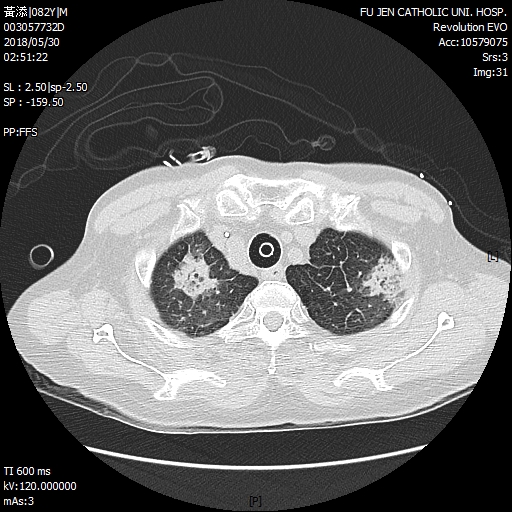

Supplement: Supplementary file 1 — Supplementary Information. [file 41598_2022_23246_MOESM1_ESM.docx]
